# COVID 19 and BAME health care staff: Wrong place at the wrong time

**DOI:** 10.7189/jogh.10.020358

**Published:** 2020-12

**Authors:** Faisal Bashir Chaudhry, Samavia Raza, Khurram Zeeshan Raja, Usman Ahmad

**Affiliations:** 1 Acute Medicine Department, Royal Stoke University Hospital, University Hospital North Midlands NHS Trust, Stoke on Trent, Staffordshire, UK; 2 Imaging Directorate, Royal Stoke University Hospital, Stoke on Trent, University Hospital North Midlands NHS Trust, Stoke on Trent, Staffordshire, UK; 3 Acute Internal Medicine, Walsall Manor Hospital, Walsall, UK; 4 Consultant Gastroenterology, Hull Royal Infirmary, Hull, UK

Of the 1.2 million staff employed by NHS, 20.7% belong to Black, Asian and minority ethnic (BAME) background. However, analysis of deaths of NHS Staff during the pandemic shows that 64% of those who died belonged to BAME background. A recent survey about this disproportionately high mortality has suggested discriminatory deployment of staff from non-white ethnic background in areas with potentially high virus exposure as a reason. We have looked at factors to explain this heavy presence of medical staff from BAME background at the front door. Moreover, we have studied mortality data related to COVID-19 and analysed it against population demographics of corresponding areas of west midlands, to highlight any trends. This has revealed a higher mortality form COVID 19 in cohorts with higher percentage of BAME population.

Of the 1.2 million employed by the NHS, 20.7% belong to Black, Asian and minority ethnic (BAME) background [1]. An earlier analysis showed that in April, out of the 119 NHS staff known to have died in the pandemic, 64% were from BAME background [2]. In a recent survey, health care staff belonging to black, Asian or ethnic minority (BAME) background were asked for their opinion about this disproportionately high mortality in NHS workers belonging to non-white ethnicities [3]. The most common reason described in the survey was deployment of BAME staff in areas with higher potential for exposure to virus. This is highly alarming and concerns like this should be addressed through a formal inquiry. If any evidence of discrimination is found in deployment of staff based on race, it could amount to one of the biggest scandals NHS has faced in recent times.

Figures released by NHS in March 2019 show that 79.2% of NHS staff are white and 20.7% belong to all other ethnic groups [1]. However, further breakdown of data suggests that majority of staff belonging to minorities take up frontline jobs [4]. So, we find a disproportionately high number of BAME staff members in medical roles, than in non-medical and managerial positions. Among those in the non-medical roles, there is even a smaller proportion reaching senior managerial positions (bands 8a to 9). Only 7% of senior managers are from BME background [5]. As a result, minority ethnic groups are systemically over-represented at lower level of NHS grade hierarchy, working in the shadow of snowy white peaks. This unequal representation remains a major problem and efforts to address this issue are under way, there is a long way to go [6]. In the current pandemic, it is possible that the higher representation of BAME on front door is due to their base job roles rather than redeployment.

A heavy presence of BAME health professionals at the front door may also be linked to the difficulties in acquiring sub-specialties positions upon arrival in United Kingdom despite having ample foreign experience. After being shortlisted for an NHS job, a white applicant is 1.45 times more likely to be successful in being appointed than applicants from minority backgrounds [7]. This difference is even higher in acute trusts, the hot spots of COVID activity [7]. The long journey of accreditation in major sub-specialities and painful hoops a specialist needs to jump through, inevitably leads to taking up posts in front door medicine. Often senior clinicians in specialties from foreign countries, take posts as junior trust grade front door clinicians while awaiting approval from GMC to go on specialist register. The same specialist register on which Europeans are eligible to be inducted into, despite difference in length of training or different language as medium of medical education.

**Figure:**
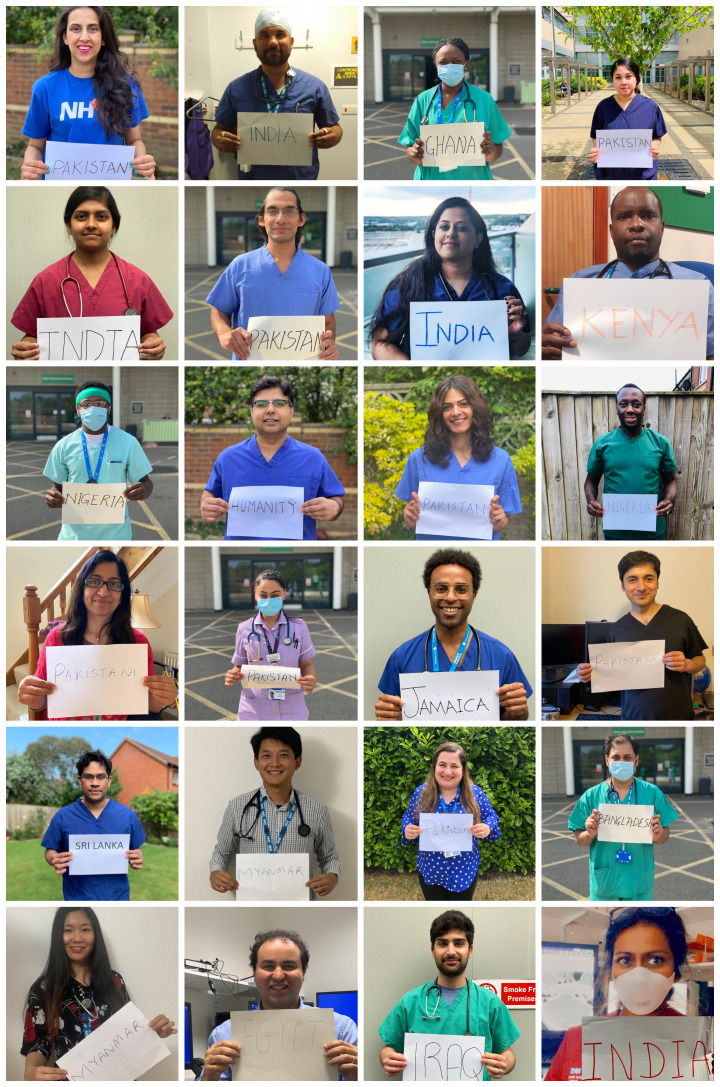
Photo: Collage of BAME health care workers in the NHS (from Faisal Bashir Chaudhry’s collection, used with permission).

There is evidence that non-white ethnicities have a high mortality from COVID 19 [8]. We have analysed mortality data of first 10 weeks of pandemic in United Kingdom, from some major acute hospital trusts across our region and compared it with patient demographics. We work in West Midlands region of United Kingdom, which has a population of 5.9 million according to 2018 mid-year estimate. 17.3% of its population belong to black, Asian and Ethnic minority background. Though, this population is not evenly distributed, in terms of both diversity and density and some areas have a higher proportion of BAME population than others. The biggest acute hospital trust in the region is the University Hospital Birmingham NHS Foundation Trust, with 2569 General and acute hospital beds, serving a population of approximately 1.3 million across Birmingham and Solihull [9]. As per 2011 census around 474 000 people from black, Asian or ethnic minority group live in this area which make up 35% of the total population. Region is one of the hardest hit areas in the UK, with 3855 cases testing positive for corona virus so far [10]. 846 deaths from COVID 19, have been confirmed in University Hospital Birmingham, making one of the highest reported mortality figures in the country reaching approximately 22%. Trend is followed in surrounding areas with high BAME population. 30% of 262 000 people living in Wolverhampton belonging to BAME background, where reported mortality is even higher. 256 deaths have been reported in Wolverhampton NHS Foundation Trust, out of a total of 956 cases testing positive for corona virus, reaching a staggeringly high mortality figure of 26.7%. Similar figures are seen across Sandwell (BAME population 28%, COVID mortality 28.4%) and Walsall (BAME Population 20%, COVID Mortality 18%) (**Table 1**).

**Table 1:** COVID-19 and BAME

Area	Main Acute NHS Trust	Population [11]	BAME Population (number) [12]	BAME population (percentage)	COVID19 Cases till 14th May 2020 [10]	Deaths till 14th May 2020 [13-15]	Mortality/case fatality
**Birmingham and Solihull**	University Hospital Birmingham NHS Foundation Trust	1 356 300	473 839	34.9%	3855	846	21.9%
**Wolverhampton**	Royal Wolverhampton NHS Trust	262 000	79 788	30.5%	956	256	26.7%
**Sandwell**	Sandwell and West Birmingham Hospital NHS Trust	327 400	92 592	28.2%	1141	325	28.4%
**Walsall**	Walsall Manor Hospital NHS Trust	283 400	56 854	20.0%	1071	192	17.9%
**Staffordshire**	University Hospital North Midlands NHS Trust	875 200	36 874	4.2%	2022	257	12.7%
**Shropshire, Telford and Wrekin**	Shrewsbury and Telford Trust Hospitals	498 100	18 481	3.7%	1006	119	11.8%
**Herefordshire**	Wye Valley NHS Trust	192 100	3304	1.7%	413	46	11%

BAME – Black, Asian and minority ethnic

Things are a different when we look at Staffordshire, where the second biggest NHS trust of the region operates. University Hospital North Midlands NHS Trust (UHNM) provides acute hospital services to approximately 900 000 people of Staffordshire and surrounding area. There have been 257 COVID-19 related deaths confirmed in UHNM in the first 10 weeks. If we compare this with the 2022 positive cases reported in Staffordshire, the mortality figure would reach around 13%. The percentage of BAME population, in Staffordshire is 4.3% which is quite low compared to neighbouring areas of West Midlands. In the population of around 500 000 living across Shropshire, Telford and Wrekin, Shrewsbury and Telford NHS Trust (SaTH) is the major hospital network providing acute hospital services. As of mid-May the trust had reported 119 in-hospital deaths. 1006 patients had tested positive for corona virus in the area. The proportion of BAME population is low at 3.7% and so is the mortality at 11.8%, when compared to the rest of the West Midlands.

A major confounding factor is the predilection of people belonging to BAME background to live in densely populated urban areas [16]. Figures from 2011 census show that 98.2% Black African, 98.7% Bangladeshis and 99.1% of Pakistanis are more likely to live in urban areas [16]. When corrected for size of population, more densely populated areas, tend to have higher number of cases. We have also observed a trend of higher mortality from COVID 19 in these areas. Cities like Birmingham, Wolverhampton and Sandwell with population density of more than 3000 people per square kilometre have a mortality figure from COVID 19 of more than 20%. On the other hand, areas like Staffordshire (population density 334 sq km), Shropshire, Telford and Wrekin (population density 142 sq km) have lower mortality figures (**Table 2**).

**Table 2:** COVID-19 and population density

	Population	Area [16]	Density per square kilometre	Cases till 14th May 2020 [10]	Cases per million [10]	Deaths till 14th May 2020	Mortality
**Birmingham and Solihull**	1 356 300	446 km^2^	3041	3855	2877	846	21.9%
**Wolverhampton**	262 000	69 km^2^	3797	956	3618	256	26.7%
**Sandwell**	327 400	86 km^2^	3806	1141	3485	325	28.4%
**Walsall**	283 400	104 km^2^	2725	1071	3779	192	17.9%
**Staffordshire**	875 200	2620 km^2^	334	2022	2310	257	12.7%
**Shropshire, Telford and Wrekin**	498 100	3487 km^2^	142	1006	1935	119	11.8%
**Herefordshire**	192 100	2180 km^2^	88	413	2149	46	11%

The higher risk to BAME population is now being recognised at senior management level in NHS Trusts and GP surgeries [17]. Risk assessment is being carried out of all staff through various scoring systems to ascertain risk to an individual health care worker. Due to paucity of available data, different health care authorities have devised their own scoring system for this purpose. The need of the hour is to develop a central standardised system to identify vulnerable members of staff and clear recommendations from NHS bodies about steps that should follow the risk assessment. Individual health care providers are taking steps at their level to protect vulnerable members of staff belonging to BAME background, which is a good start. Though mortality figures still show that health care workers belonging to ethnic minorities have been hit hard by COVID 19 and significant steps still need to be taken in this regard.
